# High Permeability Photosintered Strontium Ferrite Flexible Thin Films

**DOI:** 10.3390/mi12010042

**Published:** 2021-01-01

**Authors:** Abid Ahmad, Bhagyashree Mishra, Andrew Foley, Leslie Wood, Maggie Yihong Chen

**Affiliations:** 1 Ingram School of Engineering, Texas State University, San Marcos, TX 78666-4684, USA; abidahmad.eee@gmail.com; 2 Materials Science Engineering and Commercialization, Texas State University, San Marcos, TX 78666-4684, USA; b_m415@txstate.edu; 3 Nanohmics, Inc., Austin, TX 78741, USA; afoley@nanohmics.com (A.F.); lwood@nanohmics.com (L.W.)

**Keywords:** ferrite materials, magnetic thin films, strontium ferrite nanomaterial, relative permeability, relative permittivity

## Abstract

The paper is focused on the development and optimization of strontium ferrite nanomaterial and photosintered flexible thin films. These magnetic thin films are characterized with direct current (DC) and high frequency measurements. For photosintered strontium ferrite samples, we achieved relative complex permeability of about 29.5-j1.8 and relative complex permittivity of about 12.9-j0.3 at a frequency of 5.9 GHz.

## 1. Introduction

Ferrite materials, e.g., spinels and garnets, have attracted research interest in recent times because of their excellent microwave frequency characteristics such as low magnetic loss. Bulk ferrite materials have been widely used in the manufacture of microwave devices such as modulators, circulators, etc. [1]. However, thin films of ferrite materials have unique electrical, mechanical, and optical properties compared to their bulk counterparts. In many cases, these unique properties are a consequence of film thicknesses being below the length scale of magnetic domains in a material. As a result, desirable magnetic properties of ferrites can be engineered at the submicron level and are readily controlled. For example, the magnetic properties of zinc-ferrite thin film have been reported to show a sharp deviation from the bulk [2]. Furthermore, the permeability of a 0.6 μm a nickel-zinc ferrite thin film deposited on a 2 mm glass substrate by spin-spray exhibits a higher permeability than the Snoek’s limit of bulk Ni-Zn [3]. Ferrite magneto dielectric materials are promising for antenna miniaturization and enhanced performance at microwave frequencies [4]. The measured properties of magnetic thin films can provide deeper insight into the fundamental structure and behavior of the materials. For example, soft magnetic thin films with high permeability and permittivity at MHz and GHz frequencies have received much recent research attention [5,6] because of the rapid growth of microwave technology sectors (e.g., WiFi and 5G). There are several techniques for determining the permeability and permittivity of magnetic thin films. Some are single frequency techniques while others are techniques that provide information over a broad frequency range [7]. In this work, we investigated thin ferrite materials properties such as permeability and permittivity at both direct current (DC) and high frequencies.

DC characterization of ferrite films was considered as fundamental research. In turn, it is also important to know the characteristics of ferrite materials at high frequency. Investigation at high frequency is a possible pathway for the future application of magnetic waveguide design in the radio frequency band of interest (i.e., C-band). Nicolson and Ross [8] developed a technique for determining permittivity and permeability from measurements of the reflection and transmission coefficients of a material sample placed in a transmission line. Weir [9] applied this technique to measurements made using a network analyzer. The basic concept is to measure the S-parameters of a sample placed in a transmission line and determine the intrinsic properties required to produce these measurements.

We developed ferrite materials and deposited them on flexible Kapton films. The ferrite thin films were examined with X-ray diffraction (XRD) to observe the material crystallinity. The XRD confirmed that the ferrite thin films had good crystallinity and indicated a high-quality film. We characterized properties of the ferrite thin films under DC and high frequency measurements. The results show high magnetic permeability and electrical permittivity, which supports the XRD assessment indicating high film quality. These high-quality ferrimagnetic thin films appear promising for flexible microwave devices due to their high permeability.

## 2. Materials and Methods

Hexaferrites such as Sr- and Ba-doped hexaferrites together with bismuth ferrite, have many properties, which make them suitable for GHz devices [10,11]. We will now describe our synthesis process for the materials we studied.

### 2.1. Synthesis of Strontium Hexaferrite

**Figure 1:**
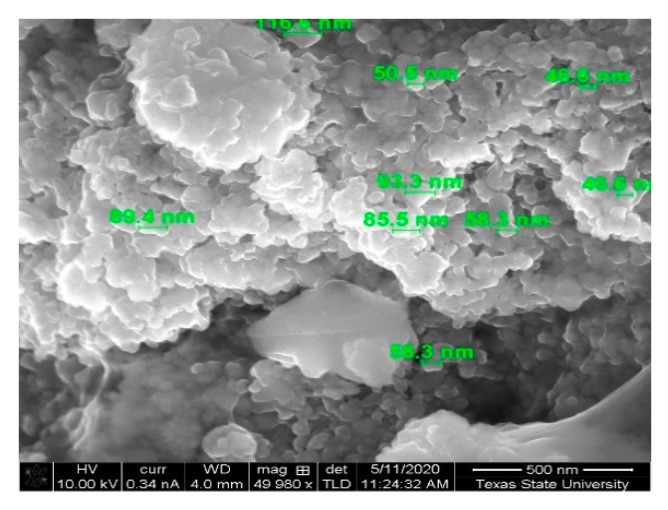
SEM of as synthesized strontium ferrite powders showing particle size between 50 and 100 nanometer. The scale bar in the lower right of the image is 500 nm.

To synthesize nano particulate strontium hexaferrite, a Pechini sol–gel chemistry was used. Iron nitrate nonahydrate and strontium chloride were dissolved in water at a 12:1 molar ratio to produce strontium ferrite hexaferrite, SrFe_12_O_19_. Citric acid and ethylene glycol were added, which undergo a transesterification reaction forming a covalent polymer network to ensnare the dissolved metal ions. To this solution, 1 M sodium hydroxide was added dropwise initiating the reaction and causing red brown solids to precipitate out of solution as the reaction became progressively basic. Enough sodium hydroxide was added to achieve a pH between 13 and 14 to ensure conversion. The precipitated particulates were collected by centrifugation and rinsed several times with water to remove the NaCl byproduct. To achieve crystallinity, the dried powder was annealed in air at 500 °C for 1 h. It is during this annealing stage where the polymeric precursor previously formed with citric acid and ethylene glycol keeps the metal ions homogeneously dispersed throughout the network and slows particle growth or “ripening” under the high temperature conditions. In Figure 1, the SEM images show the particles to be 50–100 nm and have a quasispherical appearance.

### 2.2. Paste Formulation and Application of Strontium Ferrite Thin Films

**Figure 2:**
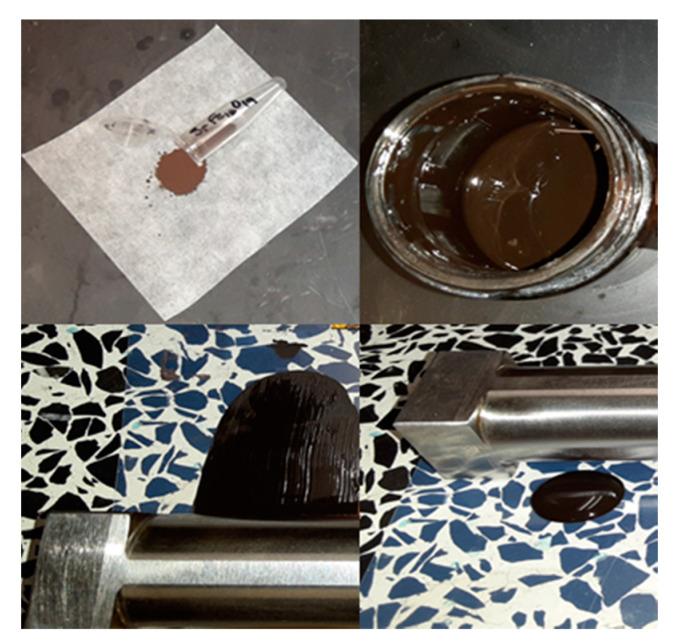
As synthesized strontium ferrite nanopowder, formulated ferrite paste and ferrite paste before and after spreading by doctor blade onto a plastic substrate.

The ferrite nanopowders were formulated into a paste consisting of terpineol (solvent), BYK 111 (dispersant), Pycal (plastisizer), polyvinyl pyrrolidone (PVP, binder) and the ferrite powder added to a mass fraction of between 60% and 80%. The mixture was then ball milled to achieve a homogeneous consistency. This paste was deposited onto Kapton and polyethylene terephthalate (PET) substrates by a simple doctor blade process. The applied films were dried in an oven at 100 °C to remove solvent. Figure 2 details the process.

### 2.3. Photosintering of Strontium Ferrite Thin Films

**Figure 3:**
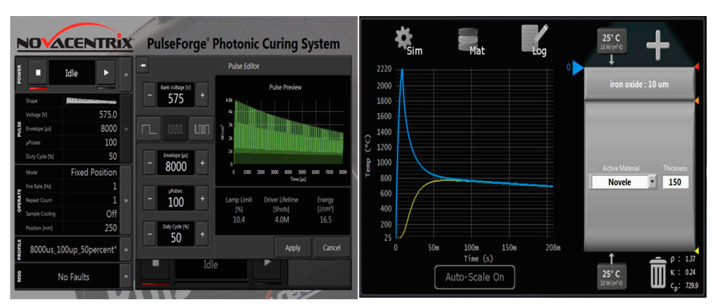
(**Left**) Screenshot of an example pulse sequence used on the PulseForge^®^ 1300 to photosinter strontium ferrite thin films. (**Right**) SimPulse^®^ predicted temperature profile for the pulse indicated.

The deposited films were photosintered with a PulseForge^®^ 1300 (NovaCentrix, Austin, TX, USA) using various voltages and pulse durations to determine the amount of incident photonic energy needed to reflow the particle grains and transform the dried particle film into a bulk material. A variety of single to multi pulse sequences with gradually increasing pulse durations up to 10 milliseconds were attempted at voltages ranging from 400 to 700 V. Longer pulse durations divided by 25–100 micropulses were found to be the most effective. An example sequence that delivers a photonic energy of 16.5 J/cm^2^ at 575 V, 8 millisecond duration, divided in 100 micropulses and 50% duty cycle, which is the percentage of time the light is on during the entire sequence is shown in Figure 3. The expected temperature peak was 2200 °C and cools to a room temperature in around one second. The PulseForge^®^ Simpulse^®^ software (NovaCentrix, Austin, TX, USA) predicts a peak temperature of 1900–2530 °C for a voltage between 525 and 700 V keeping all other parameters the same as shown in Figure 3. This corresponds to an optical energy delivered per area between 13.8 and 24.9 J/cm^2^ as the voltage increased. High temperature is required to effectively reflow the ferrite grains, which have a high melting point. Iron oxide powders are reported to have melting points between 1530 °C and 1570 °C.

**Figure 4:**
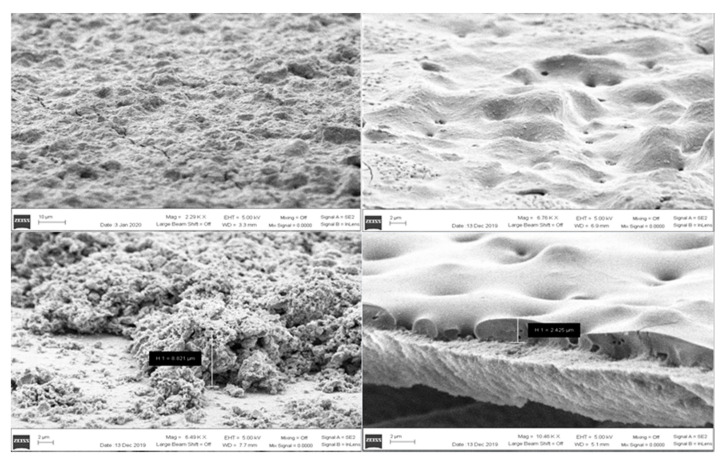
SEM images of the as synthesized strontium ferrite nanopowder film before and after photosintering. Top left and bottom left images in panel are the top-down and cross section views for the as made strontium ferrite film. Top right and bottom right images in the panel are the top-down and cross section views for the photosintered film.

For green films 10 micron thickness and below, it was found that a single sequence as shown in Figure 3 was sufficient to completely solidify, but thicker films 20 microns and above required lower voltages 450 V and for the sequence to be repeated multiple times with a few seconds between each sequence to prevent overheating of the substrate. This way the sinter can penetrate the thicker layers without melting the plastic substrates. SEM images found in Figure 4 show a deposited nanoparticle film before and after photosintering. The images on the left in Figure 4 are a top-down and cross section of the “green”, as the deposited film shown in Figure 2. The film appeared granular and a crack formed upon drying can be seen traversing the film on the top-down image. The images to the right in Figure 4 were the corresponding photosintered films. The grains were clearly reflowed as evidenced by the smooth appearance to form a bulk material. The film appeared to lose 4-6 microns of film thickness due to ablation at the surface and densification. The granular layer just below the reflowed film in the photosintered cross section image was a fumed nano silica layer that is applied to PET printing media films (Novele™) to allow the reflowed material to bind strongly to the substrate.

## 3. Results

### 3.1. DC Characterization

We characterized the magnetic and electrical properties of strontium ferrite materials at DC. The DC measurement provides data for analysis of the static properties of the films. Magnetic properties of ferrite materials were evaluated using various characterization approaches. The X-ray diffraction (XRD) was used for assessing the crystallinity of the ferrite materials. The relative permeability and relative permittivity were analyzed using a vibrating sample magnetometer (VSM) and a mercury probe station respectively. Sheet resistance of the thin film was measured by the Van Der Pauw method at room temperature and environment using the Karl Suss four-point probe station and Keysight B1500A semiconductor device analyzer that uses tungsten probes.

#### 3.1.1. XRD Analysis

X-ray diffraction (XRD) is a technique that gives us crystal structure. The intensity of the scattered X-rays was plotted as a function of the scattering angle, and the structure of the material was determined from the analysis of the location, in angle, and the intensities of scattered intensity peaks [12].

**Figure 5:**
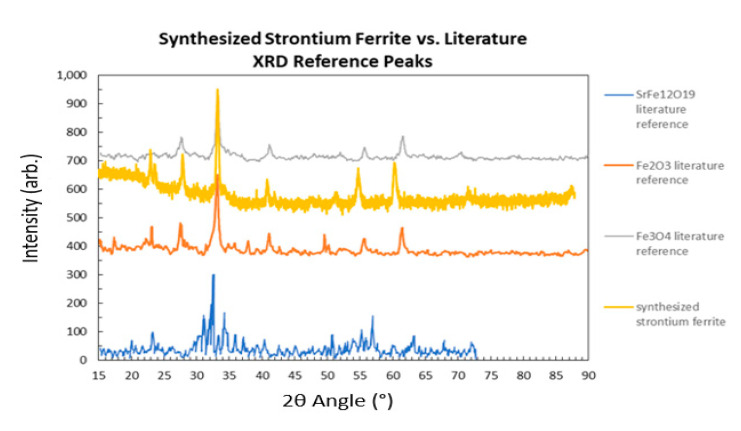
XRD analysis of synthesized strontium hexaferrite nanopowder compared to referenced XRD peaks.

Both the raw synthesized strontium ferrite (Sr-ferrite) powder annealed at 500 °C and the fabricated thin film samples (as made and photosintered using a PulseForge 1300) were analyzed by a Rigaku SmartLab X-ray diffractometer (XRD) to determine the crystalline phases. Figure 5 displays the presence of multiple peaks between 30 and 40 and 2θ confirms the introduction of an additional structural phase of the ferrite material because the known undoped ferrite phases (i.e., nano magnetite (Fe_3_O_4_) and maghemite (Fe_2_O_3_)) are both known to only yield one strong peak in this region [13,14]. Further, the primary peak from the XRD of the synthesized strontium ferrite powder revealed that the coprecipitated strontium ferrite annealed at 500 °C did not match the XRD reported in the literature for strontium hexaferrite nanopowder; instead, matching the XRD for Fe_2_O_3_ as seen in Figure 5. Further, the larger magnitude of the hump in the 15° region compared to the literature values of the hexaferrite was indicative of substantial inclusion of amorphous structure in the synthesized powder. The lattice mismatch and additional amorphous structure of the synthesized compared to the literature XRD was reasonable due to the annealing temperature (500 °C) used for the synthesized powder being substantially lower than what is reported for the literature reference (850 °C).

**Figure 6:**
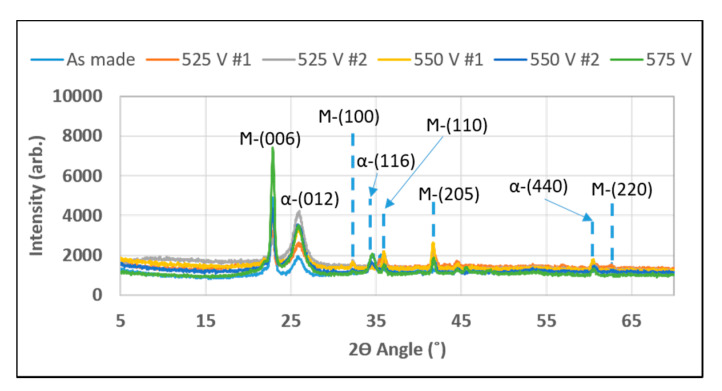
XRD analysis of as made and photosintered strontium ferrite films.

Figure 6 shows the XRD for the as made strontium ferrite film followed by two samples independently photosintered at 525 V, two samples independently photosintered at 550 V and a final film photosintered at 575 V. The same sequence was used for each photosintered film (see Figure 3) by gradually increasing the voltage, the photonic energy and peak temperature was gradually increased. The predicted temperature for these sequences was 2000 °C. Going from the as made film to photosintered, the XRD did not change peak position, however, some peaks increased in intensity. There was no clear trend as the highest and lowest intensity for the peak just below 25° (2θ angle) was reached with the lowest voltage of 525 V.

The XRD pattern in Figure 6 shows that the peaks became more intense and narrower as the voltage increased, which is an indication of an increase in crystal domain size. At the same, the diminishing height of the broad hump located at 2ϴ below 15° indicates a diminished volume of polycrystalline domains, which together with the increase in crystalline peak intensity indicates the conversion of polycrystalline volumes into crystalline ones. Given the stoichiometry of this Sr-Fe-O material system, these adjacent crystalline peaks were best identified as the 23.15° (006) peak of Sr-hexaferrite (SrFe_12_O_19_) and 24.20° (012) peak of hematite (α-Fe_2_O_3_), which would correspond to a combination of hexaferrite and hematite grains in the material. Similar inclusions of hematite impurities are observed elsewhere in the literature during Sr-hexaferrite synthesis [15].

#### 3.1.2. VSM Analysis of Permeability

A microsense LLC or EZ9-HF VSM was used to measure the permeability behavior of magnetic ferrite materials. It operates on Faraday’s law of induction, which states that a changing magnetic field will yield an electric field potential.

**Figure 7:**
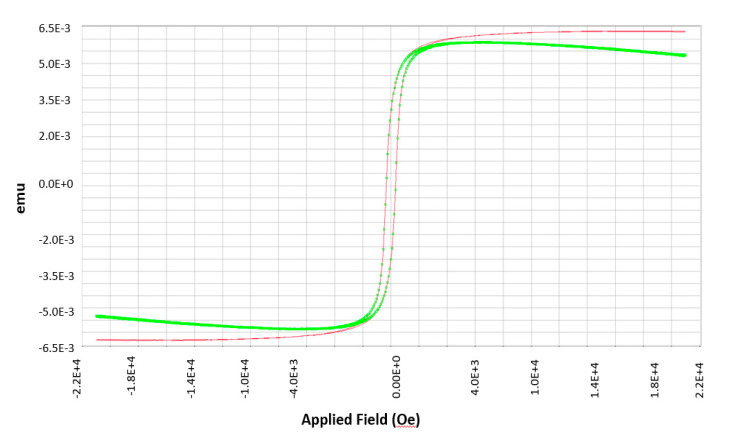
Magnetization (emu) vs. applied field (Oe).

We used the system at low temperature range from −195 to 50 °C. The sample having 7 mm length, 6 mm width, and 128.5 micron thickness was mounted on the 8 mm transverse rod of the VSM. The applied field was varied from −21000 Oersted(Oe) to 21000 Oe in the sweep mode. The measured magnetic moment in emu vs. applied field is given in Figure 7.

In Figure 7, the green colored hysteresis is as measured results from the VSM using the EasyVSM Software (MicroSense, LLC, Lowell, MA, USA). This data shows a slope after it reaches saturation. As a result, the red hysteresis is calculated by subtracting the background signal and correcting the field lag and signal slope.

**Figure 8:**
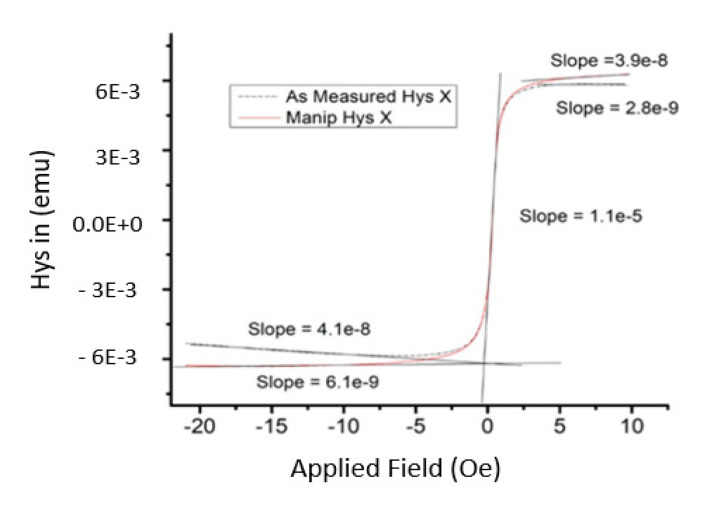
Slope calculation from the hysteresis graph.

In Figure 8, the absolute permeability was the highest slope in hysteresis loop, which was 1.10 × 10^−5^ H/m. The relative permeability was calculated using the ratio of absolute permeability and permeability of air, where the permeability of air was 1.25663753 × 10^−6^ H/m. Thus, the measured relative permeability was 8.75.

#### 3.1.3. Hermes Probe Station Analysis

The Hermes probe station is a specialized sample stage for probing thin film reactance (C, L, and R) without a top contact. Liquid mercury is applied via vacuum to a surface of the specimen and the back of the specimen was used as the other electrical contact. The thin films were deposited on silicon wafers to measures the permittivity at 0–10 MHz using this probe. The absolute permittivity and relative permittivity can be derived from $\epsilon =\frac{C\times d}{A}$ and $\epsilon_{r}=\frac{\epsilon }{\epsilon_{0}}$ where d is the thickness of the sample, area $A=4.7\times 10^{-7}$ m^2^ and $\epsilon_{0}=8.85\times 10^{-12}$ F/m. As the thin film was on Si wafer, it behaved as a MOS capacitor when connected to the Hermes probe. When DC bias voltage was applied the MOS capacitance will undergo accumulation, depletion and inversion mode. In accumulation and inversion mode charge carriers in Si substrate will be directly under the dielectric thin film, so it will give the exact capacitance (maximum capacitance in C–V curve) of the dielectric thin film. Whereas in the depletion mode, the charge carriers will be below the depletion layer; so here the total capacitance will be a series combination of both the thin film and depletion layer (minimum capacitance in the C–V curve). The relative permittivity of the Sr-ferrite film was measured using 802-150 MDC Mercury Probe station. Taking the maximum capacitance value in C–V curve with DC bias voltage from −5 to 5 V, the relative permittivity of Sr-ferrite film was found 6.48 at 1 kHz and 6.9 at 100 kHz.

### 3.2. Microwave Frequency Characterization

Since ferrimagnetic materials have promise in microwave frequency applications, we characterized the ferrimagnetic thin films within C-band (5.85–7.8 GHz) using the standard waveguide designated WR-137. The permeability was the most important parameter to define, because it governs the interaction between the electromagnetic wave and the material and was thus the origin of all magnetic phenomena. Therefore, we characterized permeability and permittivity at 5.85–7.8 GHz.

#### 3.2.1. Waveguide Signal Perturbation: Basic Principle

Many methods are available to characterize thin films at microwave frequencies. An older and simpler approach is to insert the sheet of the film into a rectangular waveguide measure the S-parameters of the system over the frequency range of interest. A segment of a rectangular waveguide where a sample has been placed, filling the waveguide and leaving no air gaps is a typical measurement configuration.

**Figure 9:**
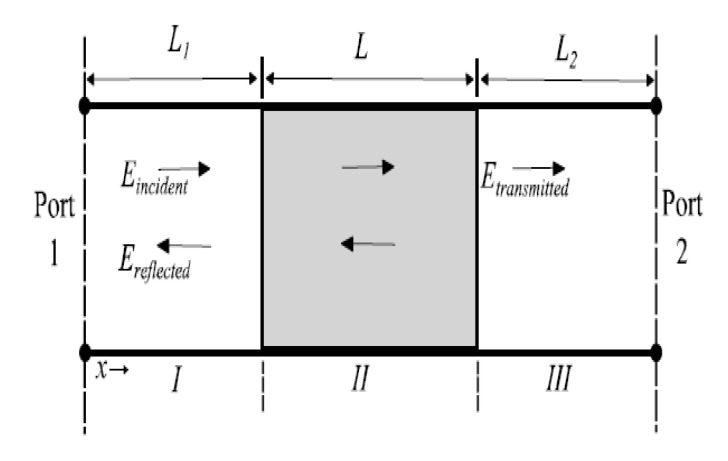
Incident, transmitted, and reflected electromagnetic waves in a filled waveguide.

Figure 9 shows such a segment whose axis is in the *x*-direction, as is the propagation direction. The electric fields at the three sections of the transmission line are $E_{I},\;E_{\mathrm{II}},\;\mathrm{and}\;E_{\mathrm{III}}$, respectively. L is the length of the under-test unit, and the total length of the waveguide can be expressed as $L_{\mathrm{Total}}$ = *L* + $L_{1}$ + $L_{2}$.

S-parameters describe the input–output relationship between ports (or terminals) in an electrical system [16]. The S-parameters of the testing structure were measured directly using a vector network analyzer and served as the input for further calculation.

##### Nicolson–Ross–Weir (NRW) Algorithm

The electric and magnetic behavior of a low-conductivity material was determined by two complex parameters, permeability, μ, and permittivity, ε. The Nicolson–Ross–Weir (NRW) algorithm [9] was used in order to calculate the permeability and permittivity of the ferrite film.

The Nicolson–Ross–Weir (NRW) algorithm combines and derives formulas for the calculation of permittivity and permeability.

(1) $$X=\frac{\left({S_{11}}^{2}-{S_{21}}^{2}\right)+1}{2S_{11}}$$

(2) $$\gamma =X\pm \sqrt{X^{2}-1}$$

where γ is a complex number, which was used to describe the behavior of an electromagnetic wave along a transmission line. γ is also known as the reflection coefficient. The appropriate sign is chosen so that γ ≤ 1 in order to express the passivity of the sample. The transmission coefficient is

(3) $$T=\frac{\left(S_{11}+S_{21}\right)-\gamma }{1-\left(S_{11}+S_{21}\right)*\gamma }$$

The complex permeability is calculated from

(4) $$\mu_{r}=\frac{1+\gamma }{\left(1-\gamma \right)*\Delta *\sqrt{\frac{1}{{\lambda_{0}}^{2}}-\frac{1}{{\lambda_{C}}^{2}}}}$$

and the complex permittivity from

(5) $$\varepsilon_{r}\mu_{r}={\lambda_{0}}^{2}\left(\frac{1}{\Delta^{2}}+\frac{1}{{\lambda_{C}}^{2}}\right)$$

where $\varepsilon_{r}$ is the relative permittivity, $\mu_{r}$ is the relative permeability, $\lambda_{C}$ is the cutoff wavelength of the transmission line section, and $\lambda_{0}$ the free space wavelength. Meanwhile, $\varepsilon_{0}\;\mathrm{and}\;\mu_{0}$ are the permittivity and permeability of the free space.

Further,

(6) $$\frac{1}{\Delta^{2}}=-{\left[\frac{1}{2\pi D}\ln\left(\frac{1}{T}\right)\right]}^{2}$$

where *D* is the thickness of sample, and $\frac{1}{\Delta }=\frac{1}{\lambda_{g}}$ where $\lambda_{g}$ is the transmission line guide wavelength [17].

Equation (6) has an infinite number of roots since the imaginary part of the logarithm of a complex quantity T is equal to the angle of the complex value plus 2πn, where n is equal to the integer of $\frac{L}{\lambda_{g}}$. The unwrapping method can be used to solve the problem of phase ambiguity [17].

This method with a network analyzer has three kinds of errors, systematic, random, and drift. Multiple measurements were taken to average out the random errors. The drift errors were minimized by the control of lab temperature and humidity. System errors were generated in the measurement setup due to coax cables, coax-to-waveguide adapters, etc. To de-embed these effects, mechanical calibration kit was used to calibrate the system to the point at the end of coax cables. The entire system without the sample was first measured and de-embed from the results with the sample inserted into the waveguides.

#### 3.2.2. Experimental Setup

**Figure 10:**
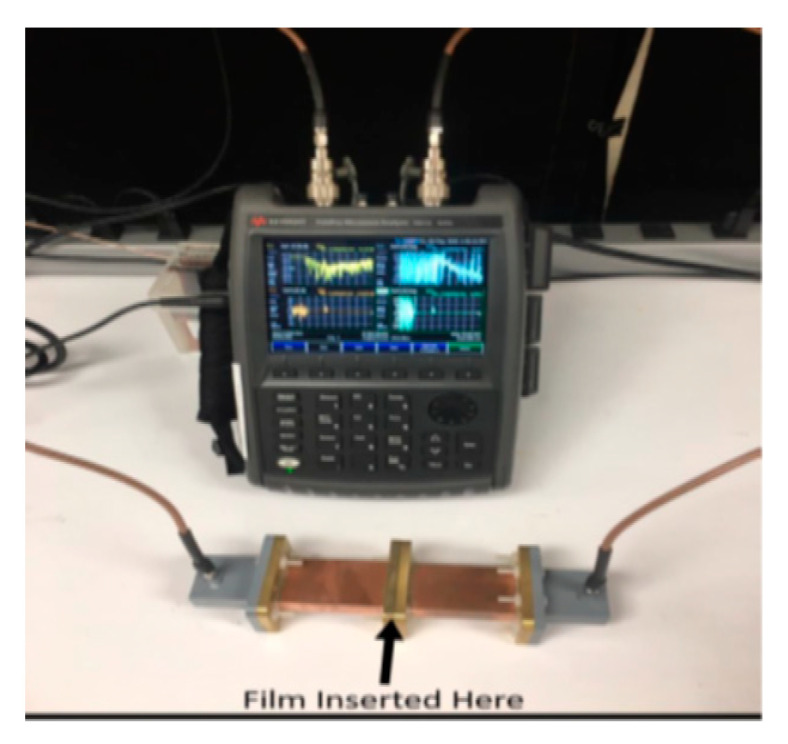
The waveguide setup for measuring thin film permeability and permittivity at 5.9 GHz. (**Top**) vector network analyzer (VNA) (**Bottom**) waveguide.

Measurements of the microwave properties of thin films were performed with the waveguide technique. Since the experimental setup involves several components such as cables and connectors, proper care has to be taken to ensure that the entire system remains stable over the measurement period. First, a bare commercial Kapton substrate was measured. The system errors were reflected and evaluated in the measured parameters of the commercial Kapton substrate. The samples examined were on a Kapton substrate one with an air-dried strontium ferrite thin film and one with a photosintered strontium ferrite thin film. The bare Kapton substrates had a thickness of 127 microns. Samples of these films were cut into a size suitable for insertion into a WR-137 waveguide shown in Figure 10 and covered the entire waveguide cavity with the film. The measurement was calibrated by normalizing to an air-filled cavity. Two independent scattering parameters S11 and S21 data were measured with a Keysight N9917A vector network analyzer (VNA) at 5.9 GHz. 

The parameters measured by the vector network analyzer were used to derive the real and imaginary parts of permittivity and permeability of the sample using the NRW algorithm. The accuracy of the constitutive material properties depends on the accuracy with which the parameters are measured.

#### 3.2.3. Experimental Results

Since the ferrimagnetic films were deposited on the Kapton film, the Kapton film was characterized first. The Kapton film was inserted between two pieces of WR-137 waveguides, which were tightened up to avoid an air gap. The S parameters were measured with the Keysight N9917A vector network analyzer after a two-port calibration. Then the measured data were plugged into the NRW algorithm program to obtain the permeability and permittivity values. The relative permeability was calculated to be 1.15 at 5.9 GHz, which gave us an error below 15%. The error mainly came from the calibration of equipment and the air gap between the waveguides. 

The strontium ferrite as-made thin film was deposited on top of Kapton. It was air dried with a thickness of 31 micron. The strontium ferrite thin film was then inserted between two pieces of WR-137 waveguides. Two samples were measured, and the values were averaged. The as-made air-dried strontium ferrite thin film had a measured relative complex permeability of 11.4-j3.6 and relative complex permittivity of 4.9-j0.9 at 5.9 GHz.

Another set of strontium ferrite thin film were photosintered right after deposition. The strontium ferrite thin film had a thickness of 22 microns. Two samples were measured, and the values were averaged. We found that the sintered strontium ferrite thin film had a relative complex permeability of 29.5-j1.8 and relative complex permittivity of 12.9-j0.3 at 5.9 GHz.

The measured data show that the photosintered strontium ferrite thin film had higher relative permeability and relative permittivity, which comes from the denser structure after photosintering.

## 4. Discussion

In summary, strontium ferrite nanomaterial was developed, and the material was deposited into thin films on flexible Kapton substrates for evaluation. DC and high frequency characterization of the strontium ferrite materials were carried out. The Sr-ferrite samples, which were photosintered, show a comparatively high relative complex permeability of 29.5-j1.8, and relative complex permittivity of 12.9-j0.3 at 5.9 GHz. In Ref. [10], powder strontium ferrite sample with an average grain size of about 50–100 µm and density 1.300 g/cm^3^ was analyzed. The transmission/reflection technique and the cavity resonators technique were used to determine the permeability and permittivity. Measurements revealed that the real permittivity of strontium ferrite powder was in the range from 2.597 to 2.712 and it had an average real permeability of 1.063. The photosintered strontium ferrite thin film demonstrated in this work had much higher permittivity and permeability. This high relative permeability and relative permittivity would make this film a strong candidate to demonstrate a possible pathway for future microwave applications such as C-band communication waveguides. These higher magnetic properties of ferrimagnetic strontium ferrite will also be suitable for antenna design and application. 

## Data Availability

The data presented in this study are available on request from the corresponding author.
